# Atrophy of retinal vessels in neovascular age-related macular degeneration following long-term treatment with 20 intravitreal anti-VEGF injections

**DOI:** 10.1186/s12886-022-02700-8

**Published:** 2022-12-05

**Authors:** Miklós D. Resch, Aniko Balogh, Tilmann Kurth, Zoltán Z. Nagy, Delia Cabrera DeBuc, András Papp

**Affiliations:** 1 Department of Ophthalmology, Semmelweis University, Budapest, Hungary; 2 Department of Ophthalmology Uzsoki Hospital, Budapest, Hungary; 3 Bascom Palmer Eye Institute, University of Miami Miller School of Medicine, Miami, FL USA

**Keywords:** Exudative age-related macular degeneration, Anti-VEGF, Long-term treatment, Optical coherence tomography angiography, Superficial retinal plexus, Deep retinal plexus, Vessel density

## Abstract

**Background:**

The study aimed to evaluate the changes in retinal vascular density in exudative age-related macular degeneration (AMD) after long-term anti-VEGF treatment using optical coherence tomography angiography (OCT-A), and to compare these changes with the vascular density in AMD treated for one year and healthy eyes.

**Methods:**

In our cross-sectional study OCT-A was performed on 60 eyes of 60 patients. Group AMD 20 × consisted of patients receiving long-term (minimum 20 injections) aflibercept therapy (*n* = 17), and Group AMD one year consisted of patients treated for one year with a treat & extend protocol (*n* = 25). The vascular density values obtained with OCT-A were compared with an age-matched control group of 18 healthy eyes. We examined the central retinal thickness (CRT), the vascular density of the fovea and parafovea in the superficial and deep retinal plexus, and evaluated the extent of the non-flow area and the foveal avascular zone (FAZ) on a 3 × 3 mm macular region. Kruskal–Wallis test was performed for statistical analysis.

**Results:**

In Group AMD 20x, the vascular density of superficial retinal plexus in the fovea (*p* = 0.0022) and parafovea (*p* < 0.0001) was significantly lower compared to Group one year and control group. In the deep retinal plexus, vascular density in the fovea (*p* = 0.0033) was significantly lower in both AMD groups compared to the control group, with no difference in the parafoveal region (*p* = 0.0774). The extent of non-flow area (*p* = 0.0003) and FAZ (*p* = 0.0008) were significantly larger in both AMD groups compared to the control group. There was a significant difference in CRT between those treated for one year and control eyes (*p* = 0.0036).

**Conclusions:**

In our study, we demonstrated that macular vessel density was lower in the foveal area in the superficial retinal plexus in AMD patients after one year and long-term anti-VEGF treatment. These vascular density changes were absent in the parafoveal and whole areas of the deep retinal plexus. Our results indicate that long-term anti-VEGF treatment reduces the vascular density of the superficial retinal plexus to a greater extent compared to the deep retinal plexus.

**Supplementary Information:**

The online version contains supplementary material available at 10.1186/s12886-022-02700-8.

## Background

The introduction of antiangiogenic therapy in neovascular age-related macular degeneration (nAMD) achieved great success in avoiding blindness for many patients worldwide [1]. Randomized clinical trials and real-life experiences have demonstrated, that intravitreal injection (IVI) of anti-vascular endothelial growth factor (anti-VEGF) can effectively and safely control choroidal neovascularization (CNV) and exudation, thus maintaining visual acuity [2]. Among the various treatment protocols, treat-and-extend (T&E) is recently recommended for the best anatomic and functional results in nAMD [3].

The latest non-invasive method of detecting retinal and choroidal vessels is optical coherence tomography-based angiography (OCT-A). OCT-A is proven to be equally non-inferior compared with invasive angiographic techniques [4] according to the REVEAL study [5], and it also provided the quantitative assessment of the macula before and after anti-VEGF treatment of nAMD [6, 7]. It has been shown, that both choroidal and retinal vascular density is affected by anti-VEGF therapy. The primary effect of antiangiogenic therapy can be seen in the gradual reduction of the central retinal thickness (CRT) and the size of CNV in the early phase of the treatment, which is beneficial for visual improvement [8].

Vascular density in the choroid decreases after anti-VEGF therapy of CNV [9], and signs of atrophy and vascular abnormalization occur [10]. OCT-A revealed that retinal vascular density does not change significantly during the loading dose, and even one year after treatment initiation, only slight retinal vascular changes are present independently of the pharmacological agent and treatment protocol [11]. However in correlation with the gradual deterioration of vision, significant atrophy of the neuroretina after long-term antiangiogenic treatment [12], retinal pigment epithelium (RPE), and choroid can be observed [7], similarly to geographic atrophy when retinal vessel density can be reduced [13].

There is limited knowledge of the quantitative OCT-A characteristics of the retinal vessels after very long-term anti-VEGF treatment in nAMD. The aim of the current study is to analyze retinal vessel parameters by quantitative OCT-A in patients, treated for nAMD with at least 20 IVI, compared with the one-year results and an age-matched control group of eyes.

## Methods

### Study Design

A total of 60 eyes of 60 subjects (30 male and 30 female) were enrolled in the retina outpatient clinic of the Department of Ophthalmology at Semmelweis University. The cross-sectional institutional study was approved by the Regional and Institutional Committee of Science and Research Ethics s (Semmelweis University No 253/2016). The study was conducted according to the tenets of the Declaration of Helsinki, and applicable national and local requirements. All patients signed written informed consent before entering the study.

### Participants

Patients with originally treatment-naive nAMD were divided into 2 groups according to the number of IVI. The Group AMD 20 × consisted of patients receiving long-term (mean number of injections: 20.29 ± 0.77) aflibercept therapy (*n* = 17). The Group AMD one year consisted of patients with nAMD treated with aflibercept for one year (mean number of injections: 6.84 ± 1.18) with a treat & extend protocol (*n* = 25). The control group (*n* = 18) was recruited from patients without macular pathology besides dry AMD without geographic atrophy (*n* = 6, AREDS Stage 1 and 2) or healthy volunteers (*n* = 12).

Ocular inclusion criteria were: clear ocular media, sufficient capability of fixation, best corrected visual acuity (BCVA) of at least 45 letters on the ETDRS chart, and scan quality of at least 6/10. Available pretreatment fluorescein angiography (FA) and OCT images for retrospective CNV evaluation: Only eyes with type 1 and type 2 CNVs were included in the study, polypoidal choroidal vasculopathy and type 3 CNV were excluded. Eyes with geographic atrophy of the macula revealed by fundoscopy, fundus autofluorescence and OCT were also excluded from the study. Further ocular exclusion criteria were: hyperopia or myopia over 6 Diopters; opaque media. Uncontrolled glaucoma, cataract surgery within 3 months, previous vitreoretinal surgery, or macular pathology, other than nAMD were exclusive.

Non-ocular exclusion criteria were any type of diabetes, uncontrolled hypertension, and systemic use of antiangiogenic drugs.

All treatments were reimbursed by the national healthcare system: intravitreal 2 mg in 0.05 ml of aflibercept (Eylea, Bayer, Germany). Aflibercept posology followed the official T&E protocol given in the Summary of Product Characteristics (SmPC) of the drug (https://www.medicines.org.uk/emc/product/2879/smpc): 3 initial monthly loading doses, and later bi-monthly injections up to 12 months extended in case of stability (the time interval to the next visit was extended stepwise by 2 weeks, in case of no disease activity based on visual acuity, OCT and fundus image). If disease activity recurred, the treatment interval was shortened, accordingly. From Month 12 to 24 T&E protocol was followed, after Month 24 T&E was changed to pro re nata (PRN) protocol.

### Imaging Protocol and Acquired Data

Comprehensive ophthalmic examination was performed on all patients, including BCVA assessment, slit lamp, and fundoscopy. OCT-A was performed in mydriasis using the AngioVue OCT-A instrument (RTVue-XR Avanti, OptoVue, Fremont, CA, USA). Scans with the highest resolution were obtained in the central 3 × 3 mm area, centered on the foveola. The superficial capillary plexus was detected automatically between the internal limiting membrane (ILM) and the inner plexiform layer (IPL); and the deep capillary plexus between the IPL and the outer plexiform layer (OPL). Potential artifacts and segmentation errors due to the distortion of the retina were manually checked in each layer.

CRT was measured in the central 1.0 mm area. Superficial and deep vessel density (VD) was evaluated in the whole image, in the central 1 mm area (fovea), and the 3 mm ring-shaped parafoveal area. Superficial non-flow area and foveal avascular zone (FAZ) areas were measured, using the built-in AngioAnalytics software (Version ReVue 2018.0.0.18) OptoVue system with an automated segmentation algorithm.

### Statistical Analysis

Groups were compared with the Fisher exact test and the Kruskal–Wallis analysis of variance and Dunn’s multiple comparison test via dedicated statistical software (GraphPad Prism, La Jolla, CA). *P*-values < 0.05 were set to indicate statistical significance.

## Results

**Fig. 1:**
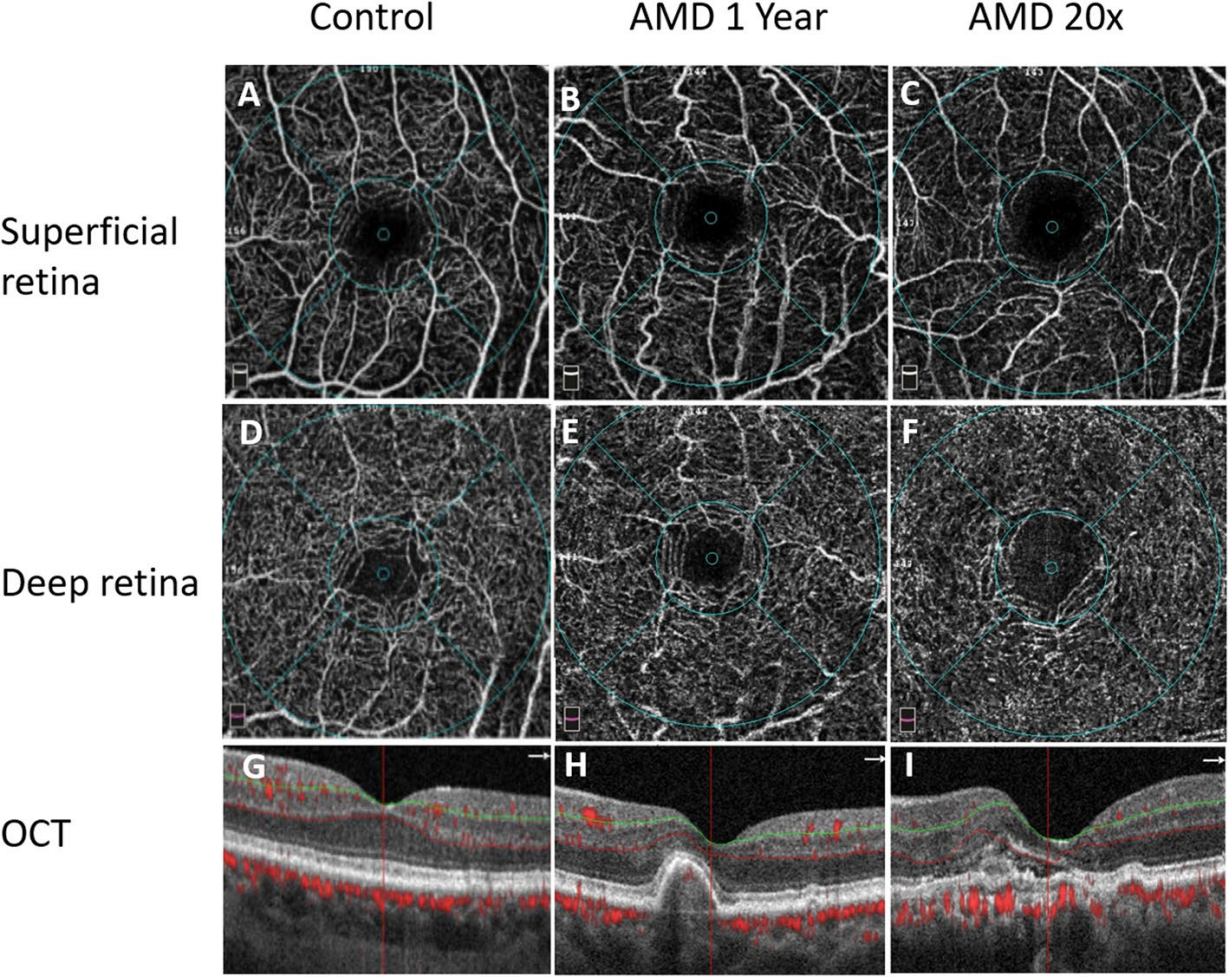
Representative OCT-A images of the groups. Superficial retinal plexus (**A**-**C**), deep retinal plexus (**D**-**F**). OCT B-scans showing the vessel cross sections in red (**G**-**I**)

Demographic and clinical data of patients are summarized in Table 1, original data S1 Table. There was no difference in age and gender in Group AMD 20 × and AMD one year from the control group. BCVA was better in the control group than in the AMD Groups as expected according to the disease under treatment. BCVA was not different in the AMD Groups. The type of CNV was not different between AMD Groups. The number of IVI and the follow-up time were higher by definition in the AMD 20 × Group (the mean duration of the treatment was 39.35 months) compared to Group one year. Representative OCTA images of al groups are demonstrated in Fig. 1.

**Table 1:** Demographic and clinical data of patients on anti-VEGF therapy and the control group. Data are shown as mean (SEM)

	**AMD 20x**	**AMD one year**	**Control**
N	17	25	18
Male/Female	12/5	10/15	8/10
Age (year)	71.12 (4.27)	74.84 (8.83)	73.33 (6.92)
BCVA (ETDRS)	63.60 (9.39)	66.00 (11.25)	82.28 (3.91)*
CNV type (1/2)	8/9	11/14	N/A
No. of IVI	20.29 (0.77)	6.84 (1.18)	N/A
Follow-up (months)	39.35 (8.24)	12.04 (0.73)	N/A

Group AMD 20x: Long-term treatment of at least 20 IVI

Group one year. One year of T&E protocol treatment

IVI- intravitreal injections

^*^p < 0.05 (Kruskal–Wallis and Fisher-exact test)

**Fig. 2:**
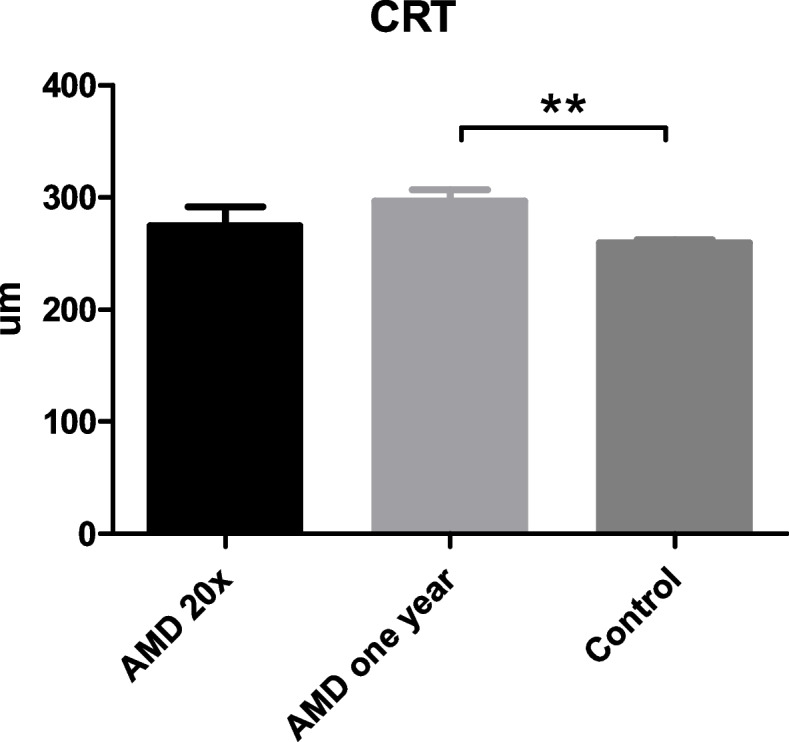
Diagram of the mean and SEM of CRT. **p < 0.01 (Kruskal–Wallis test)

Quantitative OCT-A results are summarized in Table 2. No significant difference was found in scan quality among the groups. CRT was higher in Group AMD one year compared to the controls, but none of them differed from AMD 20x (Fig. 2).

**Table 2:** Results of OCT-A evaluation: Superficial (SPF) and deep (D) vascular density in the fovea, parafovea, and the whole image. Non-flow area and foveal avascular zone (FAZ). Data are shown as mean (SEM)

	**AMD 20x**	**AMD one year**	**Control**	**p value**	**p (AMD 20 × vs AMD one year)**	**p (AMD 20 × vs Control)**	**p (AMD one year vs Control)**
Scan quality	7.0 (0.26)	7.2 (0.21)	7.78 (0.19)	0.0492	ns	ns	ns
CRT (um)	274.4 (17.39)	296.6 (9.936)	259.4 (2.701)	0.0036	ns	ns	**
SPF fovea (%)	16.82 (1.778)	25,36 (1,856)	25.67 (1.143)	0.0022	*	**	ns
SPF parafovea (%)	41.16 (1.064)	45.06 (1.067)	49.77 (1.142)	< 0,0001	*	***	**
SPF whole (%)	38.85 (1.015)	37.19 (1.159)	45.29 (0.751)	< 0,0001	ns	**	**
D fovea (%)	28.81 (1.991)	31.89 (1.386)	36.24 (0.4046)	0.0033	ns	**	*
D parafovea (%)	47.03 (1.727)	47.41 (0.914)	49.92 (0.156)	0.0774	ns	ns	ns
D whole (%)	44.24 (1.439)	45.01 (0.897)	47.31 (0.278)	0.0875	ns	ns	ns
Non-flow (mm^2^)	0.502 (0.0356)	0.504 (0.0484)	0.318 (0.0231)	0.0003	ns	***	**
FAZ (mm^2^)	0.356 (0.257)	0.349 (0.208)	0.221 (0.191)	0.0008	ns	**	**

Group AMD 20x: Long-term treatment of at least 20 IVI

Group one year. One year of T&E protocol treatment

^*^p < 0.05, **p < 0.01, *p < 0.001, ns: non-significant (Kruskal–Wallis test, Dunn's Multiple Comparison Test)

**Fig. 3:**
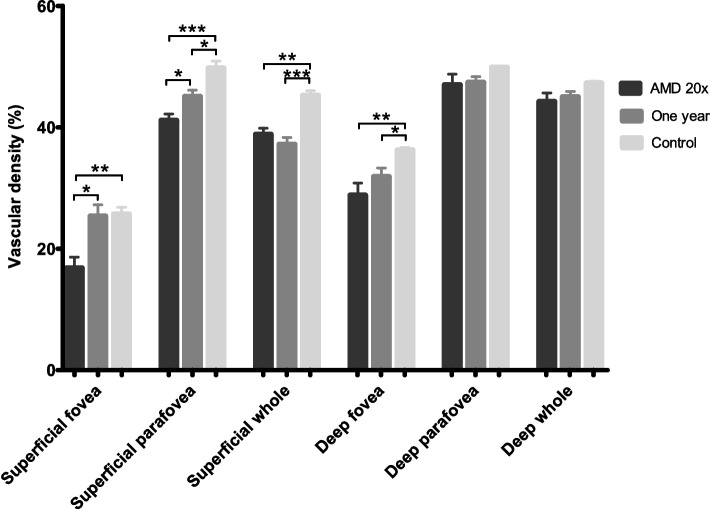
Diagram of the mean and SEM of superficial and deep retinal vascular density in all groups. *p < 0.05, **p < 0.01, ***p < 0.001 (Kruskal–Wallis test)

*Superficial vessel density*. In Group AMD 20x, the vascular density of the superficial retinal plexus in the fovea (*p* = 0.0022) and parafovea (*p* < 0.0001) were significantly lower compared to Group one year and control group. Superficial vessel density was not different between Group one year and the control group in the fovea, but vessel density was lower in Group one year compared to the control group in the parafovea and whole area (Fig. 3).

*Deep vessel density.* In the deep retinal plexus, vascular density in the fovea (p = 0.0033) was significantly lower in both AMD groups compared to the healthy control group, with no difference in the parafoveal region (*p* = 0.0774). In the deep vessel density, there was no difference between Group 20 × and Group one year neither in the fovea nor the parafovea. In the whole area, no difference could be demonstrated among groups.

**Fig. 4:**
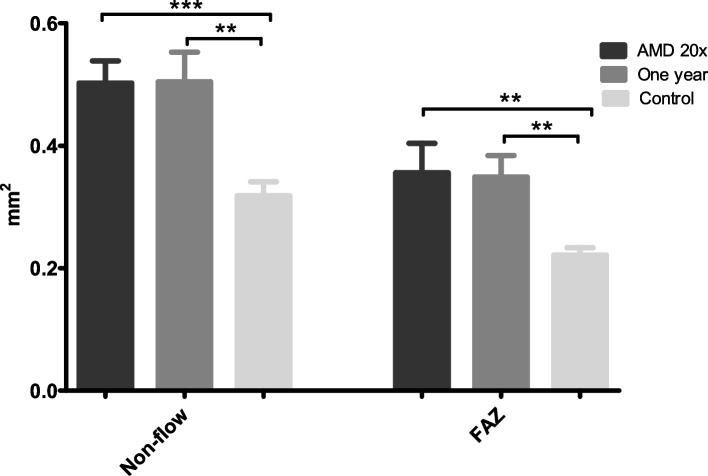
Diagram of the mean and SEM of superficial non-flow area and FAZ *p < 0.05, **p < 0.01, ***p < 0.001 (Kruskal-Wallis test)

*Non-flow area and foveolar avascular zone.* The extent of non-flow area (*p* = 0.0003) and FAZ (*p* = 0.0008) was significantly larger in both AMD groups compared to the control group (Fig. 4). No difference was found between AMD 20 × Group and AMD one year Group.

## Discussion

The effective and successful anti-VEGF treatment can maintain macular structure and visual acuity, but atrophic changes in the macula can be observed by biomicroscopy and OCT after several years of slow deterioration of visual acuity [12, 14]. The OCT-A provides structural and vascular information at the same time, thus making interpretation more complex. The time after diagnosis of nAMD and the number of treatments determine the status of the macula. The real-life, long-term follow-up study by Munk et al. [14] showed macular atrophy in 73.5% of subjects, where patients received fewer yearly injections than patients in clinical trials. One of the factors of atrophy can be the reduction of retinal circulation in the macula, which can be seen by angiography.

In our study, we compared eyes after one year of treatment and after very long-term (3.5 years at least 20 IVI) treated nAMD in order to detect changes with time. We found that retinal vascular density is more affected in the long follow-up group. However, in accordance with our previous work, some reduction in the retinal vessels can be found after one year of treatment as well [11].

There is growing knowledge on how anti-VEGF therapy affects choroidal neovascularisations in patients with neovascular AMD, but there is much less information on how anti-VEGF therapy affects retinal circulation [15].

The choriocapillaris is affected by aging, similarly to RPE. The parallel atrophy of choriocapillaris and RPE can be the background of CNV formation. When CNV is formed, some changes in the surrounding choriocapillaris can be observed, when anti-VEGF therapy is initiated, probably additional atrophy can occur in the choroid [16, 17]. The question is whether the relatively independent retinal vessels undergo changes as well or not.

The recent innovations in OCTA made it possible to measure choroidal vascular density in a reproducible way, which was found to be affected by anti-VEGF treated nAMD by Savastano et al. [18], who demonstrated choriocapillaris VD reduction in comparison to healthy eyes. Also, choriocapillaris impairment can be seen in the early phase of CNV pathogenesis, and further irregularities take place after therapy.

One year after treatment initiation, decreased retinal vascular density was seen in patients treated with a fixed T&E protocol of anti-VEGF agents compared to age-matched controls. In the foveal area, the superficial vascular density was retained, but it decreased in the parafovea; and the density of the deep retinal vessels in the fovea and the parafovea was lower than in the controls [11]. In the current study, we demonstrated additional vascular changes in long-term treated nAMD.

Our decision was not to compare 1 and 2-year results but to evaluate cases with much longer follow-up. We hypothesized that with a longer time, more pronounced changes can be detected. An earlier study by Rispoli et al. [15] investigated eyes with nAMD in the first month after a single anti-VEGF treatment with OCT-A and found that choriocapillaris vascular density fluctuation around neovascularization could be seen, which is called the dark halo.

It seems that the reduction of choriocapillary and retinal blood flow are parallel phenomena with AMD. However, it is unclear if the pathogenesis of the choriocapillaris blood flow reduction is a normal aging process, or if it is a predisposing factor for nAMD or a consequence of CNV and the anti-VEGF therapy. Arrigo et al. [6] evaluated AMD eyes before the formation of CNV and found that the reduced choriocapillary density was present in eyes even preceding the CNV, and in addition, choriocapillary porosity can be taken into account when prognosing the CNV risk.

The feasibility and the easy access to OCT-A in clinical practice might offer promising opportunities to examine the retinal and choroidal vascular density prospectively and thus we might get large data on nAMD eyes under treatment to better understand the pathophysiology of AMD in general.

In accordance with Cennamo’s findings [10], we can state that anti-VEGF therapy can be so effective in successful in the treatment of nAMD, because it significantly decreases the permeability of choroidal neovascular vessels selectively without decreasing the blood flow in the retinal and choroidal circulations. Our findings, however, point out, that after long-term treatment, the retinal vessel density still can be affected by repeated antiVEGF treatment.

In accordance with Levine [19], our previous study suggested that patients with nAMD, receiving anti-VEGF therapy have lower retinal vascular density than age-matched control subjects one year after therapy initiation, in the present study additional changes could be demonstrated after 20 injections.

There are contradictory data available on the short term effect of antiVEGF on the retinal vascular density in DME using OCTA. Mirshahi et al. concluded that there is no short term (3 months) significant effect of antiVEGF in terms of the FAZ area, or in the VD of the foveal and parafoveal superficial and deep vascular density [20]. In contrast Dabir et al. found in a similar setting that retinal vessel density decreases during 3 IVI of antiVEGF in DME [21]. There is a lack information on the very short effect of antiVEGF in wAMD eyes and we must consider the different pathomechanism of macular fluid in DME and wAMD. Both diseases are multifactorial but in DME the primary origin of intraretinal fluid is from the retinal vessel damage, and in wAMD most of the leakage originates from the choroidal neovascularisation. Thus the direct correlation between VD changes in DME and wAMD has to be performed with caution.

One limitation of our study is that in the AMD 20 × group we only included patients with relatively good visual acuity in order to achieve good scan quality, which is also reflected in the normal CRT values. Many cases with over 3 years of follow-up can have poor BCVA or atrophic macula with low CRT, which would possibly make the differences more pronounced on OCT-A. On the other hand, the automated segmentation and vessel density measurements in cases of atrophic macula usually compromise the exact quantification of vessel density. Furthermore many cases have fibrotic remnants of the original CNV, which also makes image segmentation difficult. One reason for the almost normal CRT values can be the thin subretinal scar tissue, which allows sufficient vision and fixation capability. A further limitation of the study is that we lack information on the baseline vascular density data of the eyes, before starting the treatment. At the time of the treatment start of our patients, the new software was not available yet. The relatively small sampe size can also be mentioned as a limitation of our study.

## Conclusions

In our study, we demonstrated that the vascular density of the macula was lower in the foveal area and the superficial retinal plexus in AMD patients after one year and long-term anti-VEGF treatment. These vascular density changes were absent in the parafoveal and whole areas of the deep plexus. Our results indicate that long-term anti-VEGF treatment reduces the vascular density of the superficial retinal plexus to a greater extent compared to the deep retinal plexus. With the development of OCT-A technology and new therapeutic agents novel information can be expected from further prospective studies.

## Supplementary Information

**Additional file 1.**

## Data Availability

All data are available in the supplementary material.
