# Supplementary material for: Atrophy of retinal vessels in neovascular age-related macular degeneration following long-term treatment with 20 intravitreal anti-VEGF injections
Source: BMC Ophthalmol. 2022 Dec 5;22:469. doi: 10.1186/s12886-022-02700-8 (PMC9721060; doi:10.1186/s12886-022-02700-8)
Supplement: Supplementary file 1 — Additional file 1. [file 12886_2022_2700_MOESM1_ESM.pdf]

Pat.No.	Age	Eye	VA	CNV	Scan qual	No. treat	CRT	SPF fovea	SPF parafovea	SPF whole	D fovea	D parafovea	D whole	SPF non-flow	FAZ	Follow up (Months)
1	77	OD	75	1	6	5	287	28,6	50,7	48,5	30,1	50,1	48,3	0,417	0,340	12
2	66	OS	64	2	8	8	298	27,2	45,3	34,6	30,4	49,2	47,2	0,372	0,290	12
3	83	OD	79	2	6	7	297	27,8	47,9	31,6	31,5	50,2	46,3	0,310	0,270	11
4	82	OS	64	2	7	7	321	23	46,1	34,7	33	45,3	42,3	0,526	0,430	13
5	74	OD	78	1	9	11	299	39,4	46,8	30,5	35,4	49,1	46,2	0,208	0,190	12
6	63	OD	49	1	7	8	431	39,7	49,3	31,9	40,1	44,1	42,1	0,671	0,492	12
7	85	OD	57	1	8	5	398	9	50,3	39,5	17,2	40,2	37,4	1,007	0,560	12
8	90	OD	60	1	7	6	371	16,1	43,6	36,5	23,6	39,1	37,3	1,149	0,850	12
9	77	OD	63	1	6	7	318	18,2	48,3	39,4	22,9	44,2	40,2	0,984	0,760	13
10	70	OD	69	2	8	8	293	27,2	43,3	41,6	29,5	49,4	46,7	0,599	0,430	14
11	63	OS	50	2	8	6	341	31,9	47,4	40,1	33,1	48,5	42,5	0,370	0,290	12
12	72	OD	59	2	6	6	299	28,3	50,1	32,4	30,1	46	44,1	0,321	0,270	12
13	74	OS	46	1	6	7	287	41,6	39,8	43,1	29,5	42,1	41	0,473	0,320	12
14	74	OD	70	2	7	6	199	21	45,4	25,7	27,5	42,4	40,9	0,456	0,350	12
15	83	OD	65	2	7	7	223	28,7	48,1	32,1	30,2	45,2	43,1	0,350	0,270	11
16	91	OD	76	2	6	7	297	28	49,5	33,5	34,5	42,5	40,1	0,330	0,290	11
17	85	OD	54	1	8	6	281	43,1	47,7	44,7	44,2	46,1	45,4	0,241	0,190	12
18	66	OD	59	2	8	7	279	14,8	35,4	33,3	26,6	47,7	45,3	0,559	0,206	11
19	70	OD	49	1	6	6	234	10,3	50,1	45,8	23,6	54,7	51,5	0,715	0,486	12
20	72	OD	64	1	9	7	287	21,1	39,7	38	39,6	45,8	45,3	0,477	0,220	11
21	62	OS	71	2	7	6	276	17,1	43,4	39,6	33,8	50,8	47,9	0,478	0,292	12
22	79	OS	60	2	8	7	267	30,9	34,8	34,9	48,6	54,3	52,5	0,278	0,148	13
23	84	OD	73	1	9	7	287	18	30,3	30,2	37,8	58	55,5	0,522	0,207	13
24	64	OS	70	2	7	7	267	18,1	48,2	44	28,3	49,4	47,3	0,482	0,339	12
25	65	OD	66	2	6	7	278	24,8	45	43,6	36,1	50,8	48,9	0,309	0,235	12
Mean	74,84		63,60		7,20	6,84	296,60	25,36	45,06	37,19	31,89	47,41	45,01	0,504	0,349	12,04
SD	8,83		9,39		1,04	1,18	49,68	9,28	5,33	5,80	6,93	4,57	4,49	0,242	0,172	0,73
26	67	R	69	1	9	22	334	13	35	34,7	27,4	43,1	42,6	0,461	0,258	34
27	75	L	48	2	7	20	218	25,3	42,6	42,1	43,8	53,7	50,4	0,437	0,386	37
28	72	R	65	2	6	20	324	8,8	45,9	42,6	31,2	-	47,4	0,690	0,480	28
29	77	R	60	1	6	21	238	12,7	39	35,9	28,7	53,2	49,2	0,555	0,343	42
30	68	R	74	2	7	20	222	19,9	35,5	33,3	17,4	39,6	36,7	0,493	0,428	44
31	72	L	69	2	8	20	412	21,9	46,9	45	20,2	37,3	35,9	0,339	0,318	47
32	67	L	60	1	7	20	320	15,1	43,6	40,4	23,7	43,3	41,7	0,471	0,358	50
33	68	L	69	1	6	20	192	12,9	40,5	38,4	25,1	55,8	53,1	0,585	0,475	32
34	70	L	49	2	8	20	186	18,3	32,6	29,8	29,8	38,4	35,3	0,427	0,039	34
35	71	L	85	2	6	20	189	4,4	38,6	38,4	18,7	50,9	39	0,936	0,477	33
36	75	L	48	1	6	20	281	23,9	40,5	39,4	37,1	53,5	46,8	0,431	0,290	36
37	77	R	64	1	7	20	421	26,5	45,5	42,7	33,8	34,6	35,5	0,402	0,083	40
38	67	L	69	2	6	19	265	21,2	40,9	38,1	37	47	45,8	0,475	0,223	45
39	65	L	74	1	9	20	226	25,9	48,4	45,6	37,8	49,1	47,9	0,273	0,368	39
40	66	R	60	2	6	20	248	1,6	38.0	35,1	14,9	56,1	52	0,558	0,955	60
41	78	R	86	2	8	21	276	15,8	42,8	39,6	26,7	47,6	45,8	0,520	0,290	28
42	74	R	73	1	7	22	312	18,8	43,4	39,3	36,5	49,2	47	0,487	0,278	40
Mean	71,12		66,00		7,00	20,29	274,35	16,82	41,36	38,85	28,81	47,03	44,24	0,502	0,356	39,35
SD	4,27		11,25		1,06	0,77	71,70	7,33	4,45	4,18	8,21	6,91	5,93	0,147	0,198	8,24
43	65	OS	76	0	8	0	255	17,6	38	37,8	36,3	49,9	49,3	0,405	0,233	na
44	75	OS	78	0	8	0	261	22,4	47,6	46	36,9	49	48,7	0,380	0,195	na
45	67	OS	78	0	9	0	241	19,8	45,1	42,5	36,2	49,7	48,1	0,409	0,164	na
46	78	OD	80	0	8	0	238	29,3	45,5	42,3	35,4	50,1	48,1	0,244	0,189	na
47	78	OS	81	0	9	0	267	25,9	51,6	44,1	33,2	49,6	49,1	0,307	0,201	na
48	81	OD	85	0	8	0	270	32,5	50,4	45,6	38	50	48,2	0,180	0,170	na
49	81	OS	86	0	7	0	271	22,6	47,4	44,1	35,9	48,3	46,7	0,184	0,178	na
50	65	OD	87	0	8	0	244	23,8	56	50,2	39,2	50,3	47,2	0,396	0,213	na
51	65	OS	75	0	6	0	280	20,8	47,5	44,7	37,3	51	48	0,497	0,221	na
52	67	OD	80	0	7	0	270	32,8	60,1	52,1	38,1	50,1	47,3	0,231	0,198	na
53	67	OS	85	0	8	0	261	32,1	54,5	48,1	35,1	49,9	45,2	0,237	0,194	na
54	83	OD	87	0	9	0	266	25,2	48,2	43,2	36,2	50,1	47,2	0,213	0,189	na
55	73	OD	79	0	7	0	252	20,6	52,3	47,8	33,4	51,3	46,5	0,419	0,352	na
56	77	OD	86	0	7	0	249	23,8	49,2	46,3	34,6	49,3	45,2	0,367	0,270	na
57	76	OD	85	0	8	0	269	31,4	53	46,7	39,1	49,9	46,3	0,292	0,251	na
58	65	OD	85	0	8	0	258	29,2	52,8	45,2	35,2	49,7	47,2	0,267	0,203	na
59	72	OD	84	0	7	0	263	29,7	47,9	43,3	37,1	50,1	46,3	0,249	0,221	na
60	85	OD	84	0	8	0	255	22,5	48,8	45,2	35,2	50,2	46,9	0,449	0,340	na
Mean	73,33		82,28	0,00	7,78	0,00	259,44	25,67	49,77	45,29	36,24	49,92	47,31	0,318	0,221	na
SD	6,92		3,91	0,00	0,81	0,00	11,46	4,85	4,84	3,19	1,72	0,66	1,18	0,098	0,053	na

S1 Table. The original dataset used for calculations. Groups are color coded according to “No. treat”: 6-11 AMD One year(blue), 19-22 AMD 20x (green), 0 – Control group (brown). Age is given in years at the date of examination. VA- visual acuity (ETRDS letters). CNV – choroidal neovascularization type. CRT – central retinal thickness. SPF – superficial retinal plexus. D – deep retinal plexus. FAZ – foveolar avascular zone
